# Overexpression of ABI5 Binding Proteins Suppresses Inhibition of Germination Due to Overaccumulation of DELLA Proteins

**DOI:** 10.3390/ijms23105537

**Published:** 2022-05-16

**Authors:** Ruth R. Finkelstein, Tim J. Lynch

**Affiliations:** Department of Molecular, Cellular and Developmental Biology, University of California at Santa Barbara, Santa Barbara, CA 93106, USA; lynch@lifesci.ucsb.edu

**Keywords:** abscisic acid, gibberellic acid, arabidopsis, ABI5, ABI5-binding proteins (AFPs), DELLA proteins, SLEEPY1, germination, dormancy, storage proteins

## Abstract

Abscisic acid (ABA) and gibberellic acid (GA) antagonistically regulate many aspects of plant growth, including seed dormancy and germination. The effects of these hormones are mediated by a complex network of positive and negative regulators of transcription. The DELLA family of proteins repress GA response, and can promote an ABA response via interactions with numerous regulators, including the ABA-insensitive (ABI) transcription factors. The AFP family of ABI5 binding proteins are repressors of the ABA response. This study tested the hypothesis that the AFPs also interact antagonistically with DELLA proteins. Members of these protein families interacted weakly in yeast two-hybrid and bimolecular fluorescence complementation studies. Overexpression of AFPs in *sleepy1*, a mutant that over-accumulates DELLA proteins, suppressed DELLA-induced overaccumulation of storage proteins, hyperdormancy and hypersensitivity to ABA, but did not alter the dwarf phenotype of the mutant. The interaction appeared to reflect additive effects of the AFPs and DELLAs, consistent with action in convergent pathways.

## 1. Introduction

Seed germination is regulated by diverse environmental signals, including light, temperature, and water availability (reviewed in [1]). Many of these signals affect the balance between germination inhibition by abscisic acid (ABA) and promotion by gibberellic acid (GA) [2,3,4]. Following many years of studies with exogenously applied hormones, genetic evidence for intrinsic control by this balance was provided by the isolation of non-germinating dwarf mutants that were deficient in GA synthesis [5], and germinating revertants that had additional mutations disrupting ABA synthesis [6]. In addition to control by the relative concentrations of ABA and GA, the abundance and activity of numerous signaling intermediates affect the sensitivity to these hormones.

The ABA-insensitive loci *ABI1*, *ABI2*, *ABI3*, *ABI4*, and *ABI5,* major regulators in ABA response at this stage, were identified by screens for ABA-resistant germination (reviewed in [7]). The initial *abi1-1* and *abi2-1* mutants had dominant negative mutations in members of a clade of protein phosphatases (PP2Cs) later shown to be a central part of the ABA core signaling pathway. Subsequent screens for ABA-hypersensitive germination (*AHG*) identified loss of function mutations in additional members of this clade, reinforcing the interpretation that these were negative regulators of ABA response. In contrast, *ABI3*, *ABI4*, and *ABI5* encode transcription factors that both activate ABA-induced genes and repress ABA-downregulated genes [8,9,10,11].

Screens for defects in GA response focused on plants with defects in regulating elongation, either GA insensitive dwarfs (*gai* and *gid*) [12,13] or displayed constitutive response to GA (*spindly*) [14]. The initial *gai* mutant also had a dominant negative mutation [12], reflecting the role of GAI (also known as REPRESSOR OF GA(RGA)2) as an inhibitor of the GA response. Closely related proteins include RGA1, RGA-LIKE(RGL)1, RGL2 and RGL3, and loss of function in these loci results in constitutive GA response [15]; all are members of the DELLA class of transcriptional regulators. GA response is mediated by proteasomal degradation of the DELLA repressors. Additional screens for revertants of ABA-resistance due to the *abi1-1* mutation identified *sleepy1* (*sly1*) [16], an F-box protein responsible for ubiquitination of DELLAs in the presence of GA and a functional GA receptor (GID) [17,18]. However, extended after-ripening or increased GID1 receptor expression can alleviate the extreme dormancy of *sly1* mutants by a non-proteolytic signaling mechanism [19].

Numerous direct and regulatory interactions between the ABI transcription factors, DELLA proteins, and additional transcription factors regulating germination have been reported. ABI3, ABI5, and DELLAs interact directly, apparently forming a complex in seeds exposed to high temperatures [20]. This complex activates the expression of *SOMNUS (SOM*), which encodes a CCCH-type zinc finger protein that inhibits germination by increasing ABA biosynthetic gene expression and decreasing GA biosynthetic gene expression. Under far-red light conditions, the bHLH transcription factor PHYTOCHROME INTERACTING FACTOR 1(PIF1) accumulates, also resulting in positive regulation of ABI5, DELLA proteins, and SOM, inducing expression of yet another inhibitor of germination: MOTHER-OF-FT-AND-TFL1 (MFT) [21].

Although RGA1, RGA2, and RGL2 are expressed at similar levels in dry and germinating seeds [22,23], RGL2 is reported to be the major regulator of seed germination [24,25], and its repressive function is dependent on ABI5 activity [26]. Mechanistically, this involves interactions between RGL2 and three NUCLEAR FACTOR-Y C (NF-YC) homologues (NF-YC3, NF-YC4, and NF-YC9) that synergistically bind and activate the ABI5 promoter [27]. Conversely, INDUCER OF CBF EXPRESSION1 (ICE1) interacts with both ABI5 and DELLAs to antagonize their function, thereby permitting seed germination [28].

Additional proteins implicated in antagonistically regulating the function of ABI5 and related bZIP proteins are the ABI5-binding Proteins (AFPs) [29,30]. Within this family, AFP1 and AFP2 have the strongest effects on germination. The AFPs have also been reported to regulate salt and osmotic stress through interactions with SnRK1 kinases and flowering via effects on expression of *CONSTANS (CO)*, *FLOWERING LOCUS T(FT)*, and *SUPPRESSOR OF OVEREXPRESSION OF CO(SOC)1* [31]. Proposed biochemical functions include inhibiting ABA-dependent gene expression through chromatin modifications mediated at least in part by direct interactions with TOPLESS and histone deactylase subunits [32,33] and promoting ABI5 degradation [29]. However, AFP2 overexpression promotes ABA-resistant germination that precedes ABI5 degradation [34]. The AFPs are predicted to include multiple intrinsically disordered domains, suggesting the possibility that the diversity of their interactions may reflect a role in scaffolding complexes of regulators. Given the extensive interactions already documented for ABI5, the AFPs, and the DELLAs, we sought to determine whether the AFPs also interact with DELLA proteins. We found weak direct interactions between a subset of each family in two protein–protein interaction assays, and additive antagonistic effects of overexpression/overaccumulation of these protein classes in genetic assays.

## 2. Results

### 2.1. Direct Interactions between AFPs and DELLA Proteins

**Figure 1:**
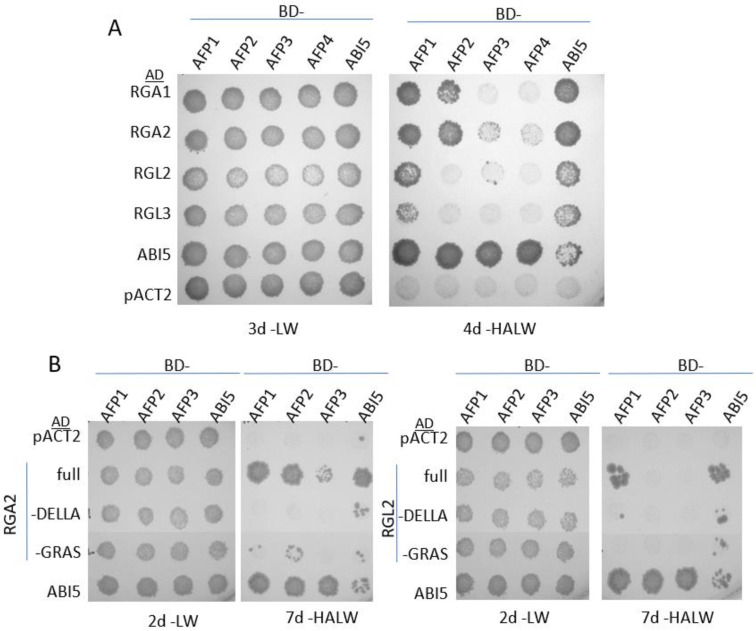
Yeast two-hybrid interactions between AFPs and DELLAs. (**A**) Interactions between the GAL4 AD alone (pACT2) or fusions to the DELLAs or ABI5 and GAL4 BD- fusions to AFPs or ABI5. (**B**) Mapping domains of RGA2 or RGL2 required for interaction with the AFPs or ABI5. Growth on -LW selects for diploids carrying both AD- and BD-fusions. Growth on -HALW requires interaction to produce GAL4-dependent activation of the HIS and ADE reporters.

We initially tested for possible direct interactions between AFPs and DELLA proteins by yeast two hybrid and bimolecular fluorescence complementation (split YFP) assays, including ABI5 as a positive control (Figure 1 and Figure S1). We focused on the DELLA proteins with similarly significant expression in dry or imbibing seeds, i.e., all except RGL1 (Figure S2). For the yeast two hybrid assays, the DELLAs were presented as GAL4 activation domain (AD)-fusions, the AFPs were fused to the GAL4 binding domain (BD), and ABI5 was present in both AD- and BD-fusions. Both AFP1 and AFP2 interacted strongly with RGA1 and RGA2/GAI, and AFP1 also interacted to a lesser extent with RGL2 and RGL3. As documented previously [20,30], ABI5 interacted directly with the AFPs and the DELLAs, especially RGA1 and RGA2. The DELLA protein interactions with the AFPs were further mapped to the GRAS domain of RGA2, but this was much weaker than the interaction with the full-length protein. Although ABI5 appeared to interact with both the GRAS and DELLA domains of RGA2, these interactions were again weaker than those with full-length RGA2.

The split YFP assays also repeated strong interactions between ABI5 and the AFPs, but weaker interactions between DELLA fusions and either ABI5 or the AFPs (Figure S1). Surprisingly, although RGL2 did not interact with AFP2 in the yeast two-hybrid assays, this combination interacted quite strongly in the split YFP assay.

### 2.2. Genetic Interactions between AFPs and DELLA Proteins

**Figure 2:**
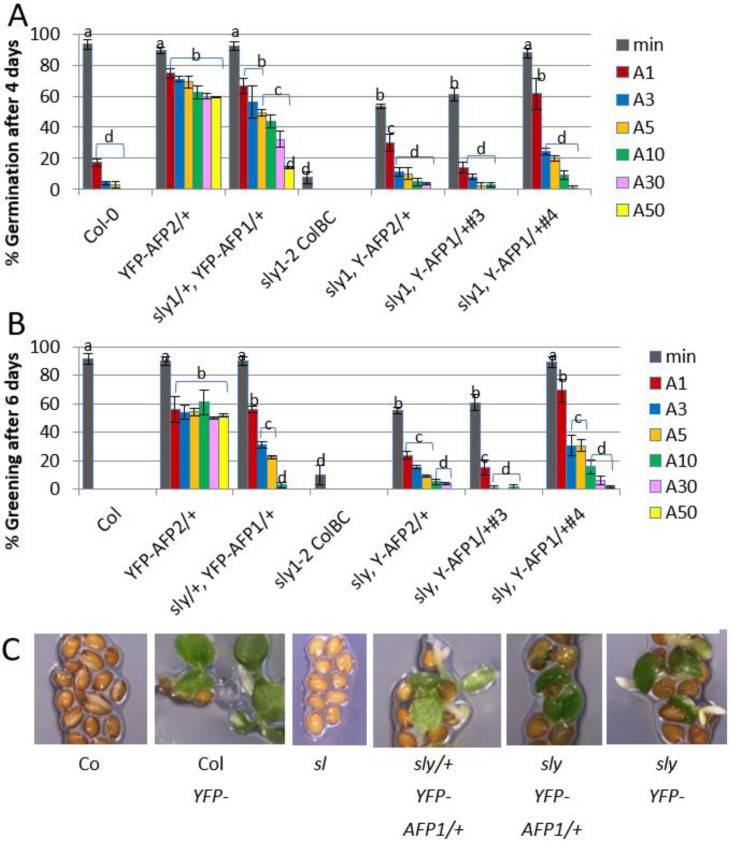
Effects of overexpressing YFP-AFP fusions in wild-type (Col-0) vs. *sly1* mutants on ABA sensitivity of seeds. (**A**) Germination of the indicated genotypes after 4 days on media with no ABA (min) or 1–50 μM ABA (A1–A50). (**B**) Greening of the germinated seedlings after 6 days on the media described in (**A**). Data displayed is the average of at least triplicate assays for each genotype and treatment + S.E. Bars with different letters represent statistically different values using Tukey’s HSD post hoc test (*p* < 0.01). (**C**) Representative images of seeds/seedlings after 6 days on 5 μM ABA.

Our previous studies showed that AFP2 overexpression results in extreme ABA resistance of seeds and consequently a loss of dormancy. In contrast, *sleepy (sly1*) mutants lack the F-box protein that ubiquitinates DELLA proteins, such that the DELLAs are hyperstable in these mutants, GA response is repressed and the *sly1* seeds are hyperdormant. To determine whether the apparent physical interactions between AFP2 and DELLAs result in epistatic or additive effects on germination potential, we overexpressed AFP2 in the *sly1-2* background. The *YFP-AFP2* transgene was crossed into *sly1* from a previously characterized line in the wild-type background [32]. Although AFP1 overexpression was less effective than AFP2 for promoting extreme ABA resistance [32], we also tested the effects of AFP1 overexpression in the *sly1* mutant because the strongest interactions observed in the yeast two-hybrid assay were between AFP1 and the DELLAs. The *YFP-AFP1* overexpression transgene from *sly1* line #3 was backcrossed into the wild-type background. As in the wild-type background, all *sly1* lines with good seedling viability were hemizygous for the transgene, such that 25% of these seed populations lacked the transgene. Overexpression of either AFP was sufficient to reduce dormancy and permit some ABA-resistant germination in this background (Figure 2A). In both backgrounds, *YFP-AFP2* overexpression promoted cotyledon expansion and greening of most germinated seeds (Figure 2B,C). However, the fraction capable of germinating, especially in the presence of ABA, was greatly reduced in the *sly1* background; a similar percentage germination was achieved for *YFP-AFP2* seeds in the wild-type background exposed to 50 μM ABA as for those in the *sly1* background on hormone-free medium. In contrast, the *YFP-AFP1* overexpression transgene from *sly1* line #3 permitted germination on media containing at least five-fold higher ABA concentrations in the wild-type background (similar responses to 50 μM vs. 10 μM ABA in wild-type vs. *sly1* backgrounds), but had more limited effects on promoting ABA-resistant greening. An independent *sly1*, *YFP-AFP1*/+ line (#4) had stronger effects in promoting both germination and post-germinative growth in the presence of ABA, but still less than the weaker transgene in the wild-type background.

**Figure 3:**
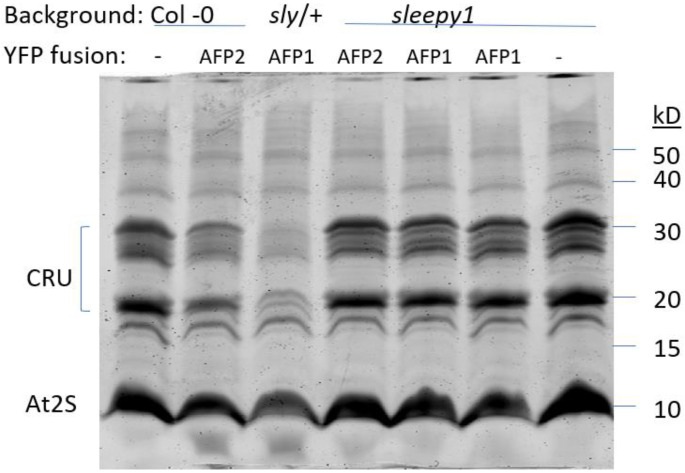
Storage protein accumulation in the indicated genotypes. Total seed protein extracts were separated on 15% SDS-PAGE and stained with Coomassie blue. Major bands represent cruciferin and At2S storage protein families.

During seed development, the ABI transcription factors mediate ABA-promoted accumulation of storage proteins and lipids. Recently, RGL3 was shown to be the major DELLA protein expressed in maturing seeds, where it promotes the expression of the cruciferin and At2S families of storage proteins [35]. Consistent with this, we found a slightly increased accumulation of these proteins in the *sly1* mutant background (Figure 3, lane 7). Transgenic lines overexpressing either AFP reduced accumulation of the storage proteins (Figure 3, lanes 2 and 3), with those in the *sly1* background again showing an intermediate phenotype (Figure 3, lanes 4–6).

**Figure 4:**
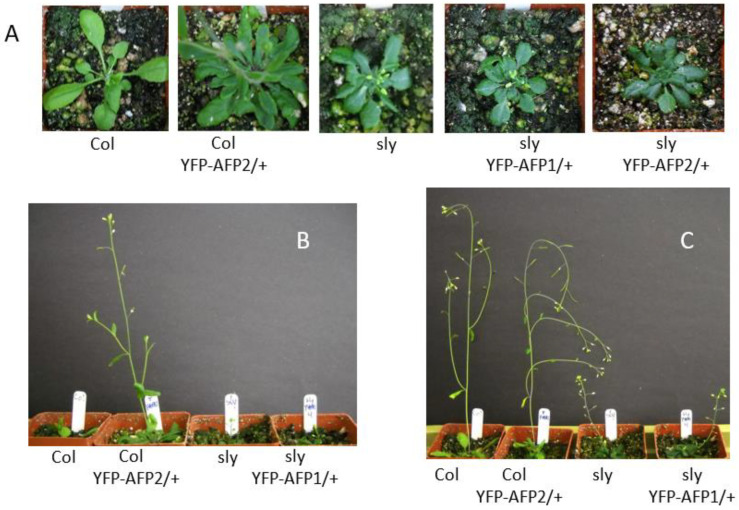
Effects of overexpressing YFP-AFP fusions in wild-type (Col-0) vs. *sly1* mutants on vegetative growth. (**A**,**B**) Five-week old plants, (**C**) seven-week old plants.

In contrast to the effects on seeds, both classes of *sly1*, *35S-YFP-AFP* lines displayed the dwarf stature, slow growth, loss of apical dominance, and poor fertility of the *sly1* parental line, although YFP-AFP2 overexpression slightly suppressed lateral branching in the *sly1* background (Figure 4 and Figure S3). Fertility was delayed the most in the primary inflorescence, with lateral shoots producing much fewer sterile flowers. Although more variable, the YFP-AFP1 transgenes enhanced the sterility in the primary inflorescence of the *sly1* parental line but did not affect fertility in a wild-type background.

### 2.3. AFP, DELLA and ABI5 Protein Accumulation

**Figure 5:**
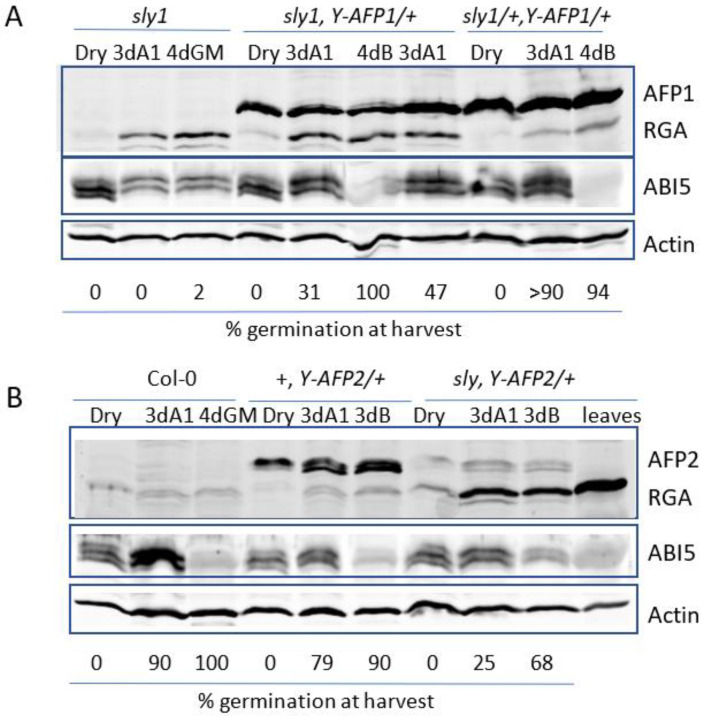
Immunoblot comparison of AFP, DELLA (RGA), and ABI5 protein accumulation in seeds and seedlings. Proteins were extracted from dry seeds or after 3d stratification, followed by 3d on GM + 1 μM ABA (3dA1) or 4d on GM, supplemented with BASTA (4dB) for the transgenic lines. Actin was used as a loading control. (**A**) Comparison of *sly1* background (lanes 1–3) with *YFP-AFP1* transgenic line #3 (lanes 4–6) or line #4 (lane 7) or the F2 from the backcross of *YFP-AFP1*#3 to Col-0 (lanes 8-10). (**B**) Comparison of wild-type (Col-0) (lanes 1–3), *YFP-AFP2* in wild type (lanes 4–6), or *sly1* backgrounds (lanes 7–10). Extract from rosette leaves is in lane 10.

ABA-resistant germination can result from decreased levels of inhibitors such as ABI5 or DELLA proteins or increased levels of proteins that antagonize their function. To determine whether the altered extent of germination was associated with changes in the levels of these proteins, we used immunoblots to compare their accumulation in seeds and following stratification in the presence or absence of added 1 μM ABA, a concentration sufficient to delay but not block germination (Figure 5). In populations segregating a YFP-AFP transgene in the *sly1* background, ABA-resistant germination was limited to individuals accumulating the fusion protein (Figure S4A).

YFP-AFP1 levels are relatively constant in seeds and seedlings, but are below detection in rosette leaves of older plants (Figure 5A and Figure S4B,C). The YFP-AFP2 fusions accumulate as germination proceeds and show a slight shift toward higher mobility, likely reflecting changes in post-translational modifications, but are also not detected in older plants (Figure 5B). Levels of the YFP-AFP fusions also varied among independent transformants, but generally paralleled the degree of ABA resistance in a given background (Figure S4C,D). However, similar amounts of the fusion protein were far less effective in promoting germination in *sly1* mutants compared to the wild-type background. For example, the YFP-AFP2 fusion protein accumulated to at least three-fold higher levels in the wild-type background than the same construct in the *sly1* background (Figure 5B and Figure S5) and permitted comparable germination on at least 50-fold higher ABA concentrations. Similarly, although the YFP-AFP1 fusion protein accumulated to similar levels in *sly1* and wild-type backgrounds, the transgene permitted a similar degree of germination on 1 μM ABA in the *sly* background as on 30 μM ABA in the wild-type background (Figure 2). Differences in YFP-AFP2 protein accumulation did not reflect different transcript levels (Figure S5), suggesting the possibility of post-transcriptional control.

The DELLA protein RGA1 is barely detectable in wild-type seeds and seedlings (Figure 5B). In contrast, RGA1 accumulates substantially post-imbibition in the *sly1* background, consistent with the roughly ten-fold increase in transcript levels reported for incubation in the presence of either water or ABA, and accumulates to even higher levels in rosette leaves (Figure 5 and Figure S4B). RGA1 levels are not substantially altered by the presence of either *YFP-AFP* transgene.

As previously reported, ABI5 levels increase post-imbibition when exposed to ABA and decrease during germination [29]. In the present study, they are mostly reflective of germination status, increasing during delayed germination of ABA-treated wild-type seeds, but showing only a slight, further delayed increase in *sly1* mutant seeds, which are not germinating at all (Figure 5 and Figure S6). In lines carrying the transgenes, germination proceeds more rapidly and by 3d post-stratification, seeds exposed to ABA are greening and ABI5 levels are not substantially higher than those in dry seeds. Similar to AFP2, ABI5 undergoes a shift in mobility in seeds incubated on media containing ABA, in this case, toward higher apparent molecular weight forms (Figure 5). ABI5 is fully degraded in all genotypes that have germinated on hormone-free media.

## 3. Discussion

ABA and GA act antagonistically throughout plant development. Plant responses to these conflicting signals are mediated through a combination of changing hormone levels and sensitivities. Levels of these hormones are controlled by environmental signals, e.g., light and temperature, and cross-regulation of hormone metabolism, while sensitivity reflects relative activities of a complex network of positive and negative regulators of response (reviewed in [2,36]). In the current work, we have focused on the interaction between two sets of negative regulators, each of which have been characterized as signaling hubs due to their extensive interactions with other signaling proteins: the DELLA repressors of GA response [37] and the AFP repressors of ABA response [32]. During seed maturation, *RGA2* and *RGL3* expression in seeds is promoted by ABI5 (Figure S2). In mature seeds *RGA1*, *RGA2*, and *RGL2* transcripts increase following imbibition with or without ABA (Figure S2) and RGL2 is subject to proteasomal degradation when GA is present, thereby permitting germination [25]. *AFP1* and *AFP2* transcripts also increase following imbibition, but are induced by ABA, high glucose and salt stress over several days, potentially acting in a feedback loop to gradually release seeds from ABA-inhibition of germination [30].

Physical interactions between subsets of the DELLA and AFP families were detected in yeast and BiFC assays in *N. benthamiana*. Although not all interactions were observed in both assays, it is not unusual to see discrepancies between their results [38]. The interactions in yeast were dependent on the GRAS domain, previously shown to be important for interactions with over 150 transcriptional regulators that can have either positive or negative effects on gene expression [39,40]. The stronger interactions observed with the full-length DELLAs might reflect contributions of additional domains not tested individually in these constructs or effects on the conformation of the GRAS domain.

Because both DELLAs and AFPs comprise families with partially redundant functions that can mask the effects of loss of function mutations, we chose to analyze interactions between the gain of function lines that over-accumulate the respective repressors. Although the use of the *sly1* background does not readily distinguish between DELLAs relevant for different developmental effects, previous studies have identified RGL2 as the major regulator of seed dormancy and germination [24], but whose function is enhanced by RGA1, RGA2/GAI, and RGL1 [15]. RGL2 acts in an ABI5-dependent manner both indirectly by increasing *ABI5* expression [26,27] and directly through complex formation [20,28]. RGL2 and several ABI transcription factors all repress *AtGASA6*, thereby inhibiting the promotion of cell wall loosening needed for cell expansion in germination [41]. RGL2 also forms a complex with DNA BINDING1 ZINC FINGER6 (DOF6) that directly activates the expression of *GATA12*, another transcription factor implicated in maintaining primary seed dormancy [42]. The Circadian clock protein REVEILLE(RVE)1 also promotes dormancy by interactions with RGL2 that stabilize it, blocking its degradation by SLY1-mediated targeting to the COP9 signalosome [43,44]. However, in after-ripened *sly1* seeds, loss of RGL2 is not required to permit germination [45].

In the present study, overexpression of either *AFP1* or *AFP2* was sufficient to release dormancy and promote some degree of ABA-resistant germination in the *sly1* background despite no ability to degrade DELLA proteins. Furthermore, ABI5 was maintained at similar levels in transgenic seeds germinating on a low concentration of ABA, regardless of whether in the wild-type or *sly1* background. This indicates that AFP-induced germination does not require loss of either the DELLAs or ABI5, and further suggests that the AFPs are acting by inhibiting the function of ABI5 and/or the DELLAs, but does not distinguish between direct and indirect inhibition. Furthermore, YFP-AFP2 appears to be subject to post-transcriptional regulation resulting in reduced accumulation in the *sly1* background. Although the mechanism is not known in this case, the YFP-AFP2 fusion is subject to proteasomal degradation [34].

While the balance between ABA and GA signaling shifts toward GA response in controlling the transition from seed to seedling at maturity, ABA signaling predominates in maturing seeds. The maize VP1 transcription factor, the ortholog to Arabidopsis ABI3, has a dual role as a positive regulator of ABA response in maturing seeds and repressor of GA response post-imbibition [8]. DELLA proteins, although initially characterized as repressors of GA signaling, can also function as positive regulators of ABA synthesis via the promotion of XERICO expression [26,46]. Increased ABA levels further promote ABA response by induction of transcription factors, including ABI5 (reviewed in [7]). During seed maturation, when GA levels are low, RGL3 is highly expressed and interacts directly with ABI3 to promote seed storage protein accumulation; overexpression of RGL3 leads to slight increases in storage protein content [35]. The *sly1* mutant has a similarly mild increase in storage proteins. In contrast, overexpression of either AFP substantially reduced storage protein accumulation, consistent with recent proteomic studies of the AFP2 overexpression line [34]; the combination of excess AFP and DELLAs was again intermediate.

All of the DELLA proteins are expressed in flowers and siliques, and genetic evidence indicates that *RGA1*, *RGL1*, and *RGL2* all regulate floral development [25]. Some aspects of DELLA regulation promote fertility: *RGA1* and *RGA2/GAI* function is required for the production of viable pollen [47] and *RGL2* positively regulate ovule number and fertility in Arabidopsis [48]. However, DELLA proteins repress cell elongation in stamens, contributing to male sterility in GA-deficient mutants [49]. Whereas loss of function for *RGA1* or *RGA2/GAI* can suppress the defects in stem elongation, apical dominance, and flowering time in *sly1* mutants, this is not sufficient to restore fertility [18]. Although YFP-AFP2 overexpression slightly reduced branching in the *sly1* background, AFP overexpression generally failed to rescue the *sly1* mutant’s growth defects.

In summary, although the mechanism is not yet clear, the *AFP* transgenes and the over-accumulated DELLAs exert additive antagonistic effects on seed development and germination. The minimal effects of the *YFP-AFP* transgenes on vegetative growth and fertility are consistent with the strong expression of RGA1 and failure to detect the YFP-AFP fusions at later stages in growth, e.g., rosette leaves. These results show a stage-specific antagonism between these two classes of signaling hubs.

## 4. Materials and Methods

### 4.1. Yeast Two-Hybrid Constructs and Assays

Full-length ORF cDNAs for RGA1 (AT2G01570), RGA2 (AT1G14920), and RGL3 (AT5G17490), (U13937, U14047, and U60167, respectively) [50] were obtained from the ABRC and an RGL2 (AT3G03450) cDNA was constructed by PCR of the coding sequence from genomic DNA, adding BglII sites to the ends using primers described in Table S1, then subcloned into the BamHI site of pUNI51. Subclones encompassing either the DELLA or GRAS domains of RGA2 and RGL2 were constructed by PCR using the full-length cDNAs as templates and primers as indicated (Table S1), and ligated into pUNI51. The RGA2 DELLA domain clone included codons 1–121, the RGA2 GRAS domain clone included codons 115–533, RGL2 DELLA domain clone encoded amino acids 1–145, and the RGL2 GRAS domain included codons 139–547. Fusions between the GAL4 activation domain (AD) and these DELLA clones were constructed by recombination with pACT2lox, catalyzed by GST-CRE as described in [51]. The GAL4 binding domain (BD) fusions to the AFPs and both AD- and BD-fusions for ABI5 have been described previously [32]. Interactions were tested by matings between pairs of haploid lines carrying BD- and AD-fusions, replica-plated onto selective media to test for activation of the HIS3 and ADE2 reporter genes, as described in [32].

### 4.2. Plant Materials and Transgenes

Arabidopsis plants were grown in pots in growth chambers under continuous light at 22 °C. *SLY1* loss of function lines was described in [16]; we used a line carrying the *sly1-2* allele that had been backcrossed three times into the Col-0 background. The *35S:YFP:AFP* fusions and split YFP fusions for ABI5, AFP1 and AFP2 were described in [32]. The 35S:YFP:AFP2 line used was #4A2. *Agrobacterium tumefaciens*-mediated direct transformation of *sly1-2* mutants was performed by the floral dip method [52], followed by the selection of BASTA-resistant seedlings. Essentially all germinating seeds were transgenic, reflecting suppression of the strong dormancy of *sly1* mutants. Progeny continued to segregate BASTA-resistance over multiple generations. A *35S:YFP:AFP1* transgene was backcrossed from the *sly1* background into Col-0 and F1s screened for BASTA resistance; within the F2 population, germination is limited to approximately 94% of seeds that have a wild-type *SLY1* allele and/or the transgene.

Split YFP fusions for RGA, RGA2, and RGL2 were constructed using the Gateway compatible pSITE-cEYFP-C1 (Acc# GU734652) vector and PCR products with attL ends added as described in [53], using primers described in Table S1, following manufacturer’s instructions for LR Clonase reactions (Invitrogen). BiFC assays were conducted as described in [32].

### 4.3. Plant Growth Conditions

Germination assays testing ABA sensitivity of age-matched seeds were performed on minimal nutrient media supplemented with ABA at concentrations over the range from 0–50 µM, as described in [32]. Following sterilization and plating, plates were incubated 3d at 4 °C; this was sufficient to break residual dormancy for all except the hyperdormant *sly1* parental line. Accumulation of fusion proteins was assayed by immunoblots of seeds or seedlings harvested after 3–4d incubation on Germination Medium (GM: 0.5 × MS salts and vitamins, 1% sucrose) with or without 1 µM ABA or 8 µg/mL BASTA, solidified with 0.7% agar.

For analysis of adult plant development, seedlings were transferred to soil and photographed at weekly intervals. The number of shoots was scored for individual mature plants (n = 11−22), and the number of sterile pods on the main inflorescence and at least 4 lateral branches of each plant was counted.

### 4.4. Protein Analyses

Seeds or seedlings were ground directly in 1× or 2× Laemmli loading buffer, respectively, microfuged 10 min at 4 °C to pellet debris, then boiled 5 min prior to fractionation by SDS-PAGE (10% polyacrylamide). Proteins were transferred to nitrocellulose filters, as described in [32]. Filters were blocked with Casein blocking buffer (LI-COR Biosciences, Lincoln, NE, USA), then co-incubated with anti-GFP mAb (1:10,000, UBPBio, Aurora, CO, USA) and anti-RGA pAb (1:2000, AS11 1630, Agrisera, Vännäs, Sweden) primary antibodies, followed by anti-mouse and anti-rabbit secondary IRDye 800 conjugated IgGs, and visualized using the 800 channel of the Licor Odyssey Infrared Imaging System or the iBright FL1500 Imaging System (Invitrogen, ThermoFisher Scientific, Waltham, MA USA). Filters were subsequently probed with anti-ABI5pAb (1:10,000, Ab98831, AbCam, Cambridge, UK) and anti-actin mAb (A0480, Sigma, St. Louis, MO, USA), followed by anti-mouse secondary IRDye 800 conjugated IgGs (LI-COR Biosciences, Lincoln, NE, USA).

### 4.5. Transcript Analyses

RNA was extracted, as described in [32], then incubated with RQ1 DNase (Promega, Madison, WI, USA) and RNAsin for 15 min at room temperature. The reaction was stopped by the addition of EGTA (1.8 mM final), then RNA was purified over Zymo-Clean columns (Zymo Resesarch, Irvine, CA, USA) according to the manufacturer’s instructions. Approximately 0.5 ug of RNA was used as a template for cDNA reactions using MMLV reverse transcriptase or GoScript (Promega, Madison, WI, USA) and a 10:1 mix of random hexamers and oligo dT as primers. cDNA concentrations were assayed by qRT-PCR using EvaGreen Master Mix (Midwest Scientific, Valley Park, MO, USA) or Forget-Me-Not EvaGreen Master Mix (Biotium, Fremont, CA, USA) in an iQ5 cycler (BioRad, Hercules, CA, USA) according to the manufacturer’s instructions. Primers used for normalizing were selected for uniform expression in seeds and seedlings grown in a variety of conditions [54]. Reactions with both primer sets were quantified relative to a standard curve spanning the range of concentrations present in all samples, as described in [55].

## Data Availability

Transcriptome data from Nakabayashi et al. (2005), the Yamaguchi lab, and Schmid et al. (2005), is present on the Arabidopsis eFP browser at (bar.utoronto.ca) [22,23].

## Supplementary Materials

The following are available online at https://www.mdpi.com/article/10.3390/ijms23105537/s1.
