# Supplementary material for: Overexpression of ABI5 Binding Proteins Suppresses Inhibition of Germination Due to Overaccumulation of DELLA Proteins
Source: Int J Mol Sci. 2022 May 16;23(10):5537. doi: 10.3390/ijms23105537 (PMC9144539; doi:10.3390/ijms23105537)
Supplement: Supplementary file 1 [file ijms-23-05537-s001.zip › ijms-1674594-supplementary.pdf]

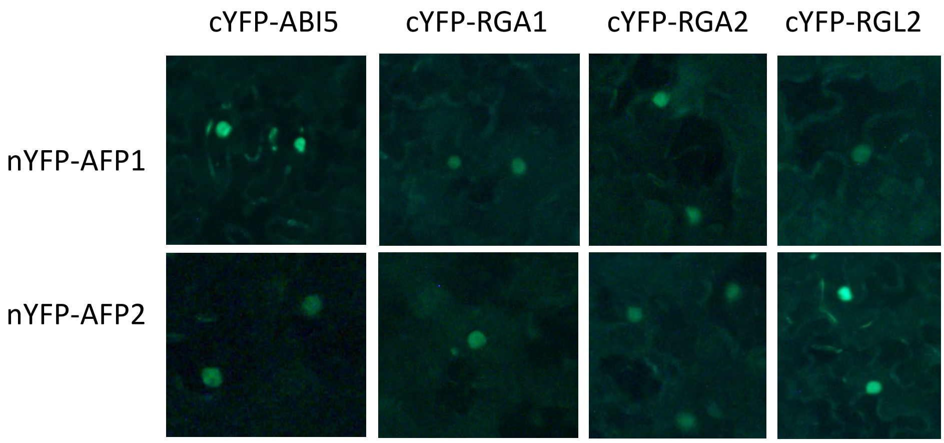

Figure S1. Interactions between AFPs and ABI5 or DELLA proteins assayed by bimolecular fluorescence complementation. Agrobacteria carrying plasmids encoding the indicated pairs of fusions to the N-terminal (nYFP) or C-terminal (cYFP) portions of yellow fluorescent protein were co-infiltrated into *N. benthamiana* leaves. Micrographs of the lower epidermis were taken 2-3d after infiltration.

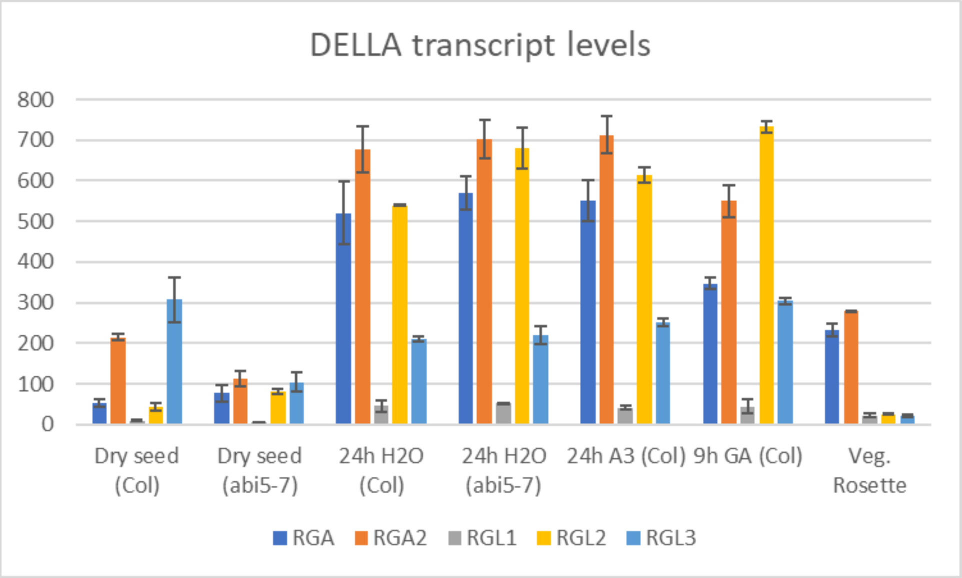

Figure S2. DELLA transcript levels in dry and imbibing seeds and vegetative rosettes. Transcriptome data from Nakabayashi et al 2005, the Yamaguchi lab, and Schmid et al 2005, present on the Arabidopsis eFP browser at (<bar.utoronto.ca>) [22, 23].

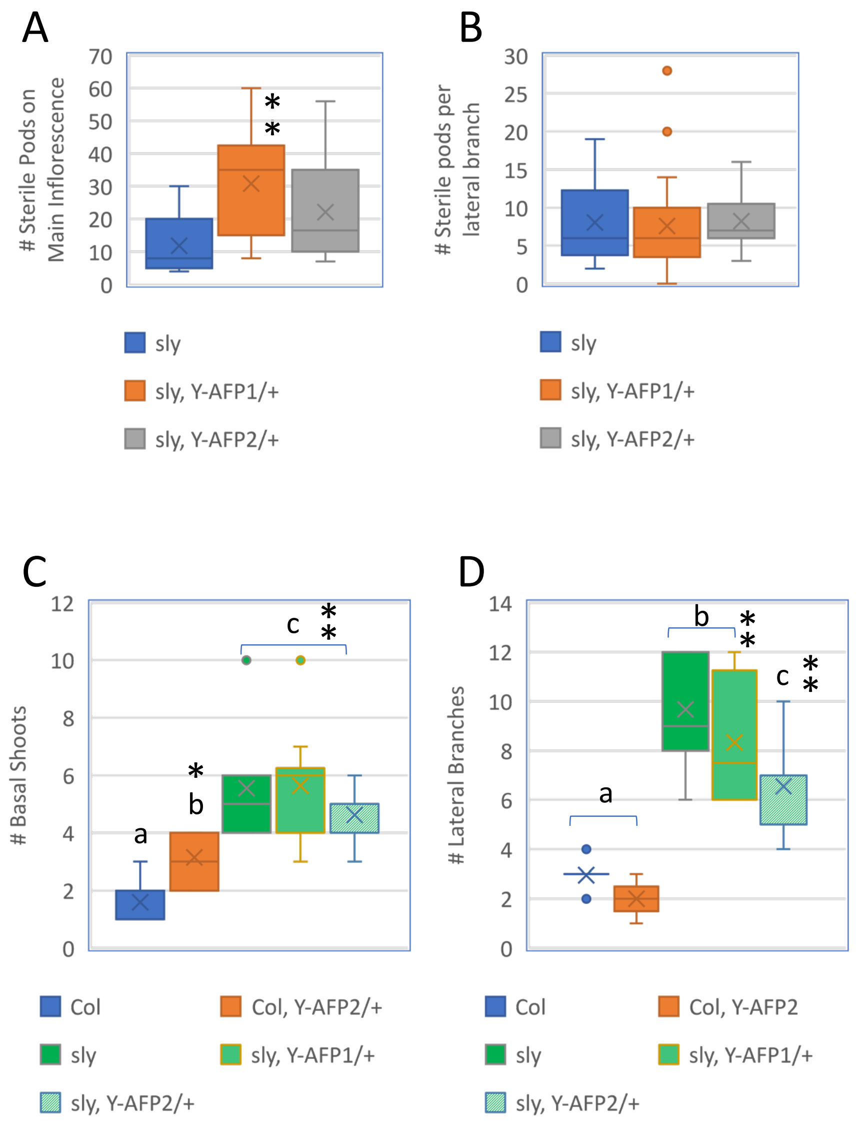

Figure S3. Reduced fertility and apical dominance of the *sly1* mutants, with or without the YFP-AFP transgenes. (A) Number of sterile pods in main inflorescence before production of fertile flowers. No sterility was observed in the Col-0 background. (B) Number of sterile pods per lateral branch. (C) Number of shoots originating from rosette. (D) Number of lateral shoots per plant. Graphs display data distribution with mean (X), first and third quartiles within the box. Statistical significance was determined by OneWay Anova with post-hoc Tukey HSD [52]. * = P<0.05, ** = P<0.01 relative to *sly1* (panels A & B) or Col (panels C & D).

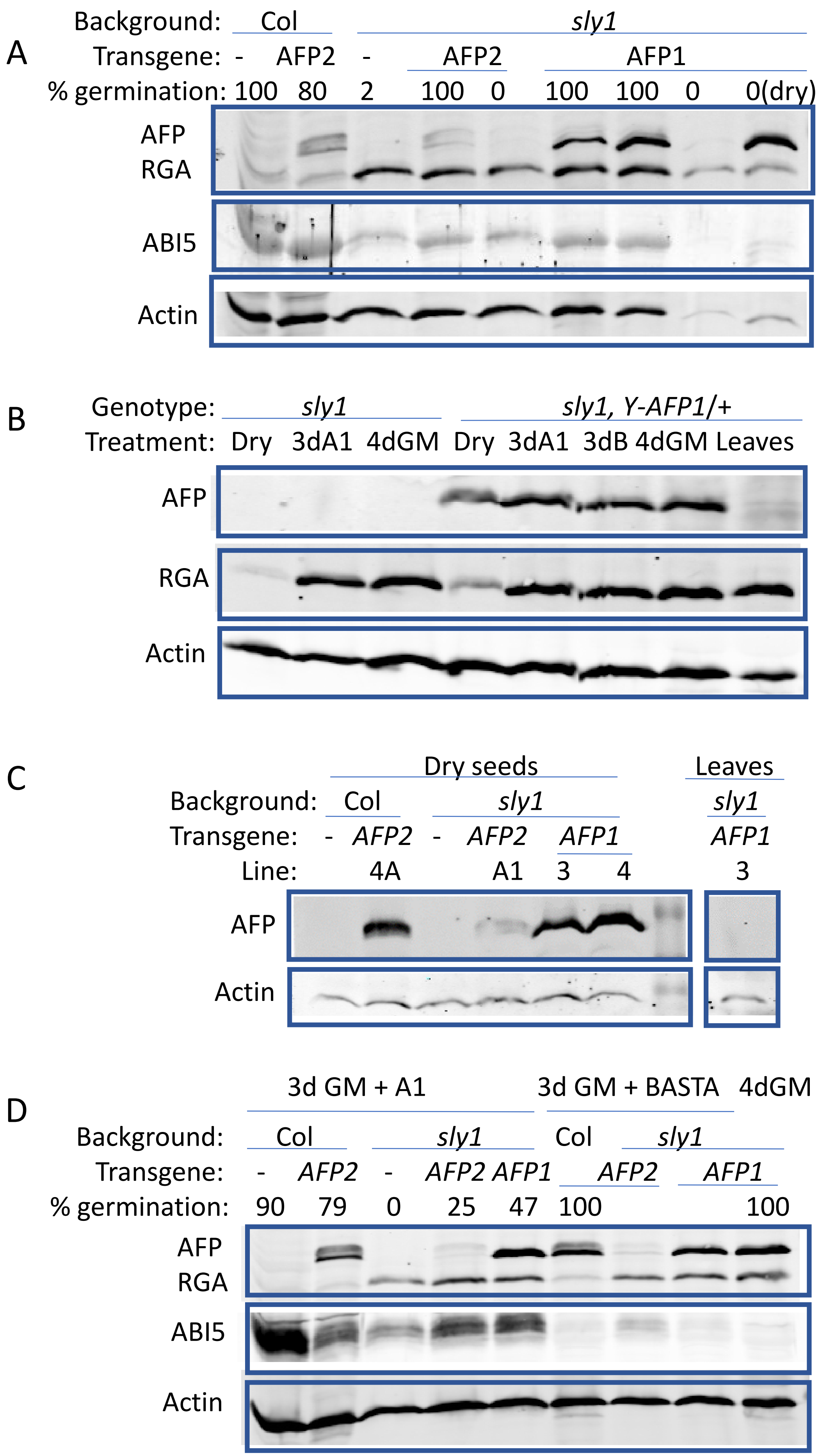
A
Background: Col sly1
Transgene: - AFP2 - AFP2 AFP1
% germination: 100 80 2 100 0 100 100 0 0(dry)
AFP
RGA
ABI5
Actin
B
Genotype: sly1 sly1, Y-AFP1/+
Treatment: Dry 3dA1 4dGM Dry 3dA1 3dB 4dGM Leaves
AFP
RGA
Actin
C
Dry seeds Leaves
Background: Col sly1 sly1
Transgene: - AFP2 - AFP2 AFP1 AFP1
Line: 4A A1 3 4 3
AFP
Actin
D
3d GM + A1 3d GM + BASTA 4dGM
Background: Col sly1 Col sly1
Transgene: - AFP2 - AFP2 AFP1 AFP2 AFP1
% germination: 90 79 0 25 47 100 100
AFP
RGA
ABI5
Actin

Figure S4. Immunoblot comparisons of AFP, RGA, and ABI5 accumulation in all genotypes at specific time points. Actin was used as a loading control. (A) Comparison of YFP-AFP, RGA, and ABI5 accumulation in seeds or seedlings after 4d incubation on GM or dry seeds (lane 10). The YFP-AFP transgenes are segregating in all backgrounds. For those in the sly1 background, separate extracts were made from germinated and non-germinating seeds. YFP-AFP expression was limited to the germinating seeds. (B) Comparison of RGA and YFP-AFP1 accumulation in *sly1* mutants with or without the *YFP-AFP1* transgene in dry seeds, leaves or after 3d incubation on GM with either 1 μM ABA (A1) or BASTA (B) or 4d on GM with no supplement. (C) Comparison of YFP-AFP fusion protein accumulation in dry seeds of the indicated lines or rosette leaves of sly, YFP-AFP1 #3. (D) Comparison of YFP-AFP, RGA, and ABI5 accumulation in seeds or seedlings after 3d incubation on GM with either 1 μM ABA, BASTA or no supplement (lane 10)

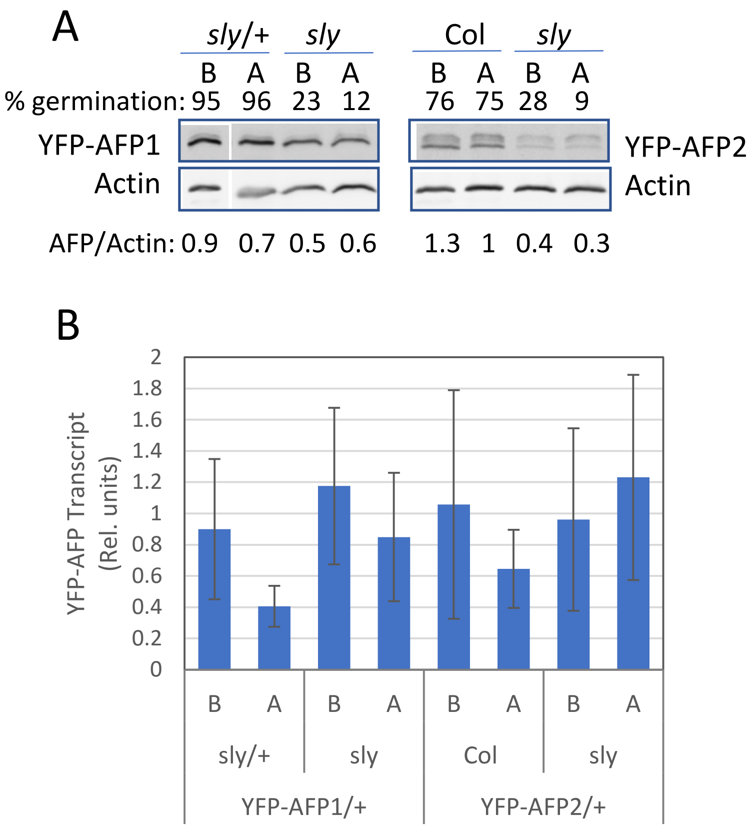

Figure S5. Comparison of YFP-AFP protein and transcript accumulation in wild-type and *sly1* backgrounds. Seeds and seedlings were harvested after 3d on GM with either 1 μM ABA (A) or BASTA (B). (A) Immunoblot comparisons of representative samples. AFP:Actin ratios were determined from scans of replicate blots with duplicate samples and a standard dilution series. (B) Relative levels of YFP-AFP transcripts in samples harvested in parallel to those used for protein determination. YFP-fusion transcripts are expressed relative to PP2AA3 transcript, previously identified as relatively constant in seeds and seedlings. Differences in transcript levels were not statistically significant.

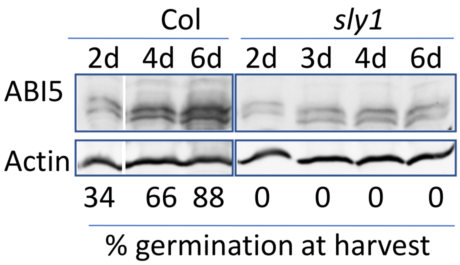

Figure S6. Timecourse of ABI5 accumulation post-stratification in wild-type (Col) and *sly1* mutants. Seeds were incubated on GM with 1 μM ABA for the indicated days post-stratification before harvest and extraction.

Table S1. Primers used for construction of cDNA clones and subclones, and qRT-PCR.

Primer name	Sequence
BglII-RGL2-F	G AAA GAT CTT ATGAAGAGAG GATACGGAGA AAC
BglII-RGL2-R	G AAA GAT CTCA GGC GAG TTT CCA CGC C
BglII-Stop-RGL2-DELLA-R	G AAA GAT CT tca CTCGTCAAAGACTTCCTCTTCC
BglII-RGL2-GRAS-F	G AAA GAT CTT GAAGAGGAAGTCTTTGACGAG
Bam-stop-RGA2-DELLA-R	CGCGGATCC tca CGAAGAAGCCGAATCGATAG
EcoRI-RGA2-GRAS-F	CCGGA ATT CCTATCGATTCGGCTTCTTCGT
attL1-RGA2	GCTcctgcaggACCATGAAGAGAG ATCATCATCA TCATC
attL2-RGA2	GAAAGCTGGGTctcgagCTA ATT GGT GGA GAG TTT CCA AG
attL1-RGA1	GCTcctgcaggACCATGAAGAGAG ATCATCACCA ATTC
attL2-RGA1	GAAAGCTGGGTctcgagTCA GTA CGC CGC CGT C
YFPqRT-F	TCAAGATCCGCCACAACATC
YFPqRT-R	TGGTAGCTCAGGTAGTGGTT
PP2AA3qRT-F	TAACGTCCGCAAAATGATGC
PP2AA3qRT-R	GTTCTCCACAACCGCTTGGT
